# Associations Between Dietary Habits and Sleep Duration in Japanese Children: The Fukushima Health Management Survey

**DOI:** 10.1111/ped.70565

**Published:** 2026-10-06

**Authors:** Enbo Ma, Tetsuya Ohira, Shuntaro Itagaki, Itaru Miura, Hirooki Yabe, Rie Mizuki, Naoko Horikoshi, Masaharu Maeda, Hideto Takahashi, Michio Shimabukuro, Mitsuaki Hosoya, Seiji Yasumura

**Affiliations:** ^1^ Health Promotion Center Fukushima Medical University Fukushima Japan; ^2^ Department of Epidemiology Fukushima Medical University School of Medicine Fukushima Japan; ^3^ Radiation Medical Science Center for the Fukushima Health Management Survey Fukushima Medical University Fukushima Japan; ^4^ Department of Neuropsychiatry Fukushima Medical University School of Medicine Fukushima Japan; ^5^ Department of Disaster Psychiatry Fukushima Medical University School of Medicine Fukushima Japan; ^6^ Medical Statistics Unit, Department of Pharmaceutical Sciences Teikyo Heisei University Tokyo Japan; ^7^ Department of Diabetes, Endocrinology, and Metabolism Fukushima Medical University School of Medicine Fukushima Japan; ^8^ Department of Pediatrics Fukushima Medical University School of Medicine Fukushima Japan; ^9^ Department of Public Health Fukushima Medical University School of Medicine Fukushima Japan

**Keywords:** adolescent, children, dietary habit, Fukushima Health Management Survey, sleep duration

## Abstract

**Background:**

Studies on the impact of dietary habits on sleep in children remain inconsistent. The study aimed to evaluate associations between dietary habits and sleep duration in Japanese children.

**Methods:**

Dietary habits and sleep time questionnaire data were available for 28,429 children from the Fukushima Health Management Survey, 2014–2019. Five dietary habit questions were asked of children aged 1–15 years, four additional questions of those aged 4–15 years, one additional question of those aged 7–15 years, and one additional question of those aged 13–15 years. Accordingly, dietary habit scores (score‐5, score‐9, score‐10, and score‐11) were calculated for the corresponding age groups. Linear mixed models were used to examine associations between dietary habits and sleep duration.

**Results:**

In the cross‐sectional analysis, the coefficients (95% confidence intervals [CIs]), expressed as hours per score unit, for dietary habit score‐5, score‐9, score‐10, and score‐11 with sleep duration were 0.018 (0.012–0.025), 0.018 (0.011–0.025), 0.018 (0.011–0.024), and 0.024 (0.010–0.038), respectively. In the longitudinal analysis, associations of changes in score‐5, score‐9, and score‐10 with changes in sleep duration were no longer statistically significant after multivariable adjustment. Among students aged 13–15 years, each one‐point increase in score‐11 was associated with higher odds of adequate sleep time (odds ratio, 1.06; 95% CI, 1.02–1.09).

**Conclusions:**

Healthier dietary habits may be associated with longer sleep duration in Japanese children; however, the longitudinal findings did not provide sufficient evidence of an independent association over time.

## 1 Introduction

Sleep duration and sleep quality in children have declined over several decades [1]. Short sleep duration can increase the risk of obesity in children, particularly those aged 3–13 years [2, 3]. Sleep disturbance is also a risk factor for cardiovascular abnormalities in healthy adolescents [4]. A Canadian study showed that longer sleep duration (> 9 h of nighttime sleep) was associated with healthy adiposity levels in young children.

Previous studies reported positive associations between the intake of fruits, vegetables, and milk and sleep duration, and negative associations between the intake of sweets, snacks, and fast food and sleep duration [5, 6, 7]. High‐glycemic carbohydrate intake may trigger compensatory hyperinsulinemia and subsequent counter‐regulatory hormone responses, which could contribute to nocturnal arousal [8]. Dietary components may influence sleep through several pathways, including modulation of systemic inflammation and oxidative stress, effects on serotonin and melatonin synthesis [9], and metabolic effects involving glycemic control [10].

Conversely, short sleep duration may increase the risk of obesity by increasing ghrelin and decreasing leptin levels, thereby leading to an increase in appetite and higher food consumption [11, 12]. It should be noted that community, family, and individual factors, including food availability and cost, can influence dietary intake and behavior [13]. Ineffective parenting strategies may underlie children's access to unhealthy foods, which, in turn, may contribute to poor sleep [14]. Parents play a crucial role in supporting children's psychological development and fostering healthy lifestyle habits, including food preferences and eating behavior [13].

Asian adolescents' bedtimes were later than those of their peers from Western countries, resulting in reduced sleep duration [15]. As school grade increased, Japanese children had later bedtimes, and sleep duration decreased by 2.0 h across the nine grade levels [15, 16]. According to the Brief Child Sleep Questionnaire, 18.3% of children had sleep disorders, and 30%–40% exhibited sleep symptoms at bedtime, during sleep, and at wake time [16].

Following the Great East Japan Earthquake and the Fukushima Daiichi Nuclear Power Plant accident in 2011, many residents were evacuated from affected areas. The Fukushima Health Management Survey (FHMS), which monitors the long‐term health of residents [17], reported that short sleep and oversleeping in children were associated with mental health problems assessed using the Strengths and Difficulties Questionnaire (SDQ) [18, 19]. Among adults with evacuation experience, mental health was associated with dietary habits after the earthquake [20]; however, this relationship has not been well examined in children. Therefore, evacuation experience and mental health are important factors to consider when examining the association between dietary habits and sleep duration in children affected by the disaster.

Evidence on the association between overall dietary habits and sleep duration in children remains limited, particularly, from longitudinal studies. In this study, we hypothesized that dietary habits in children may be associated with sleep duration. Thus, this study aimed to assess the association of dietary habits with daily sleep duration using cross‐sectional and longitudinal analyses of data on Japanese children from the FHMS.

## 2 Materials and Methods

### 2.1 Study Participants

The FHMS, comprising 210,189 individuals in the target population, was designed to monitor the long‐term health of residents living in the area affected by radiation disclosure following the Fukushima Nuclear Power Plant accident, which occurred in the aftermath of the Great East Japan Earthquake on March 11, 2011 [17]. The government specified the evacuation zone based on the spatial radiation dose and the distance from the nuclear power plant. Residents in Hirono‐machi, Naraha‐machi, Tomioka‐machi, Kawauchi‐mura, Okuma‐machi, Futaba‐machi, Namie‐machi, Katsurao‐mura, and Iitate‐mura, as well as parts of Tamura City, Minami‐Soma City, Kawamata‐machi, and Date City, were defined as evacuees. Residents in areas outside the 30 km radius from the nuclear power plant, including the rest of Tamura City, Minami‐Soma City, Kawamata‐machi, and Date City, were defined as non‐evacuees [21]. A total of 28,759 children aged 1–15 years who completed the annual mental health and lifestyle survey, a part of the FHMS, between July 2014 and December 2019, were included in this study. The parents or grandparents of children under 12 years answered the questionnaire, and junior high school children partially self‐administered. Details of the study protocol and implementation for the mental health and lifestyle survey, including dietary habits and sleep time in children, were described elsewhere [17, 22].

The study was conducted in accordance with the ethical principles outlined in the Declaration of Helsinki. The ethics review committee of Fukushima Medical University approved the study protocol (#Ippan 2020‐239). All participants' parents or legal guardians provided written informed consent prior to the surveys being conducted. This study did not involve any patient or public involvement. However, after the survey, study teams provided telephone counseling and offered necessary support to participants who needed assistance for mental health or lifestyle issues based on their responses.

### 2.2 Sleep Duration

The total daily sleeping time of the children was recorded in hours and minutes within 24 h [18]. In addition, the question on self‐assessed adequate sleep time, “Do you have adequate daily sleep time (adequate, slightly inadequate, and inadequate)?” was only for adolescents aged 13–15.

### 2.3 Dietary Habits

The children's dietary habit questions in the mental health and lifestyle survey of the FHMS were designed to monitor children's long‐term healthy dietary behavior [17, 18]. Eleven questions on the dietary habits of children aged 1–15 years were asked based on age group. Questions in younger age groups were included in older age groups. Questions one to five were on dietary intake of food groups for all children; other questions were on eating behavior for children ≥ 4 years, including Question 10 (eating breakfast daily) for school children and Question 11 (sleep within 2 h after dinner) for junior high school children. Table S1 shows four age groups' questions and answer codes.

The answer to each dietary habit question was yes or no, and each participant was assigned a score of one for a healthy habit and zero for a poor habit. The sum of scores was calculated according to the answered questions, that is, score‐5 (range 0–5), score‐9 (range 0–9), score‐10 (range 0–10), and score‐11 (range 0–11), according to age groups 1–15, 4–15, 7–15 (elementary and junior high‐school age), and 13–15 (junior high‐school age) years, respectively (Table S1).

### 2.4 Covariates

Data on the characteristics of children, including their current health status (very good, good, normal, and poor), physical activity (every day, two to four times a week, once a week, and none for children aged ≥ 2 years), and body mass index (BMI, kg/m^2^), calculated as body weight (kg) divided by height in meters squared, were available for each survey year. The mental health status of children aged ≥ 4 years was assessed using the Strengths and Difficulties Questionnaire (SDQ) and the parents' overall judgment of children with difficulties in emotion, concentration, peer relations, or activity (no/yes). The study participants were classified as evacuation and non‐evacuation areas (no/yes). The SDQ is a 25‐item questionnaire used to identify emotional and behavioral difficulties in children. It comprises five subscales: emotional symptoms, conduct problems, hyperactivity/inattention, peer relationship problems, and prosocial behavior. Higher scores indicate greater emotional and behavioral difficulties [18].

### 2.5 Statistical Analysis

Participants with a total daily sleep time (*n* = 330) of < 4 or > 20 h were excluded from the analysis. Finally, 28,429 were included in this study. The data on covariates were > 95% complete. Multiple imputations were applied using the fully conditional specification techniques to account for missing data on dietary habit questions under the assumption of missing‐at‐random. Approximately 0.02%–4.2% of missing data were imputed (missing data specified in Table S2). Body mass index standard deviation score (BMI‐SDS) was calculated using the LMS method with age‐ and sex‐specific Japanese BMI reference functions reported by Kato et al. [23]. Because only completed age in years was available, age in months was approximated by the midpoint of each completed‐year interval.

Linear mixed models were used to analyze the associations between dietary habits and daily sleep duration from 2014 to 2019, accounting for repeated measurements within individuals. An unstructured covariance structure was specified for repeated measures. Model 1 was adjusted for age (category) and sex (boy/girl) for all children; Model 2 was further adjusted for survey year (2014–2019), BMI‐SDS (continuous), current health condition (very good, good, or normal/poor), physical activity (every day, two to four times a week, once a week, or none), and evacuation area (no/yes) for children aged ≥ 2 years; Model 3 was further adjusted for SDQ (continuous) and overall mental health problems (no/yes), measured at each survey year for children aged ≥ 4 years. This analysis mainly evaluated the overall cross‐sectional associations between dietary habits and sleep duration using annual survey observations, including children who responded to only one annual survey (*n* = 4907).

For the longitudinal analysis, linear mixed models were used to estimate associations between changes from baseline in age‐specific dietary habit scores (independent variable) and changes from baseline in sleep duration (dependent variable) among children with repeated observations [24, 25]. Children with more than one observation who were younger than 15 years at baseline were included. Models were adjusted for baseline dietary habit score, baseline age, baseline sleep duration, follow‐up time, and the other covariates used in the cross‐sectional analysis, where applicable. Repeated observations within children were accounted for using a compound symmetry covariance structure.

To assess the association between dietary habit scores and self‐assessed adequate sleep time among junior high school students, a logistic regression model was used with adequate sleep as the event and adjustments made in the aforementioned approaches.

In addition, sensitivity analysis was conducted using the same regression approaches for complete case analysis without imputing dietary habit variables and covariates. In particular, stratified analyses by evacuation experience and overall mental health problems in children were also conducted using the imputed data.

The nonparametric and χ^2^ tests were used to compare differences among variables, and the least‐squares means and standard errors (SEs) of sleep duration were calculated. Regression coefficients, odds ratios (ORs), and 95% confidence intervals (95% CIs) with probability were reported. SAS 9.4 (SAS Institute Inc., 2023) was applied for all data analyses.

## 3 Results

The baseline characteristics of children and adolescents in the 2014 survey were displayed across age groups (Table 1). The means of dietary habit scores for groups of children were 2.7–3.0, 5.4–5.8, 6.3–6.6, and 6.4 for score‐5, score‐9, score‐10, and score‐11, respectively, and were more likely to decline with increasing age. Approximately < 3.7% of children had poor health conditions. BMI increased with age, and preschool children had more frequent physical activities than school children. Approximately 40% of the children were from the evacuation areas.

**TABLE 1:** Sample characteristics of Japanese children in the Fukushima Health Management Survey 2014 (*n* = 6646).

	Children aged 1–3, *n* (%)	Children aged 4–6, *n* (%)	Children aged 7–12, *n* (%)	Children aged 13–15, *n* (%)
Total	1073 (100)	1449 (100)	2850 (100)	1274 (100)
Sex				
Boy	544 (50.7)	720 (49.7)	1452 (50.9)	651 (51.1)
Girl	529 (49.3)	729 (50.3)	1398 (49.1)	623 (48.9)
Dietary habit scores (range), mean (SE)				
Score‐5 (0–5)	3.0 (0.04)	2.9 (0.04)	2.8 (0.03)	2.7 (0.04)
Score‐9 (0–9)	—	5.8 (0.04)	5.7 (0.03)	5.4 (0.05)
Score‐10 (0–10)	—	—	6.6 (0.03)	6.3 (0.05)
Score‐11 (0–11)	—	—	—	6.4 (0.05)
Physical activity^a^				
Everyday	417 (53.0)	783 (54.0)	299 (10.5)	592 (46.5)
2–4 times/week	236 (30.0)	447 (30.9)	903 (31.7)	171 (13.4)
Once a week	74 (9.4)	135 (9.3)	678 (23.8)	105 (8.2)
None	60 (7.6)	84 (5.8)	970 (34.0)	406 (31.9)
Health status				
Very good	374 (34.8)	444 (30.6)	783 (27.5)	316 (24.8)
Good	459 (42.8)	597 (41.2)	1161 (40.7)	426 (33.4)
Normal	227 (21.2)	389 (26.9)	865 (30.4)	485 (38.1)
Poor	13 (1.2)	19 (1.3)	41 (1.4)	47 (3.7)
Body mass index (kg/m^2^), mean (SE)	16.1 (0.09)	16.0 (0.07)	17.5 (0.07)	19.5 (0.11)
SDQ^b^, mean (SE)	—	17.8 (0.13)	17.8 (0.09)	17.2 (0.14)

Abbreviations: SDQ, Strengths and Difficulties Questionnaire; SE, standard error.

^a^ Available for children aged ≥ 2 years.

^b^ Available for children aged ≥ 4 years.

The average total sleep durations were 11.5, 10.2, 8.8, and 7.4 h in children aged 1–3, preschool, elementary school, and junior high school students, respectively. The sleep durations varied in each survey year. However, declining trends in sleep duration as age increased were consistent in each year's survey (Table 2). Sleep duration by age and age group, together with the corresponding recommendations, is shown in Table S3. Schoolchildren generally slept less than recommended by the guidelines.

**TABLE 2:** Daily total sleep duration based on age groups and survey years in Japanese children according to the Fukushima Health Management Survey 2014–2019 (*n* = 28,429).

	All	2014	2015	2016	2017	2018	2019
*Sleep duration*							
1–3 years							
*n*	4390	1073	923	779	677	487	451
Mean (SE)	11.5 (0.05)	11.5 (0.03)	11.5 (0.03)	11.5 (0.03)	11.6 (0.04)	11.6 (0.04)	11.5 (0.05)
4–6 years							
*n*	5402	1449	1359	911	716	510	457
Mean (SE)	10.2 (0.85)	10.2 (0.02)	10.1 (0.02)	10.1 (0.03)	10.2 (0.03)	10.3 (0.04)	10.3 (0.04)
7–12 years							
*n*	12,752	2850	2714	2177	2007	1579	1425
Mean (SE)	8.8 (0.04)	8.9 (0.01)	8.9 (0.01)	8.8 (0.01)	8.9 (0.01)	8.8 (0.02)	8.8 (0.02)
13–15 years							
*n*	5885	1274	1325	967	856	724	739
Mean (SE)	7.4 (0.26)	7.2 (0.03)	7.1 (0.03)	7.1 (0.03)	7.6 (0.03)	7.6 (0.04)	7.7 (0.04)
All							
*n*	28,429	6646	6321	4834	4256	3300	3072
Mean (SE)	9.2 (0.05)	9.3 (0.02)	9.2 (0.02)	9.2 (0.02)	9.3 (0.02)	9.2 (0.03)	9.1 (0.13)
*Adequate sleep time* ^a^							
13–15 years							
*n* (%)	2565 (43.6)	549 (43.1)	602 (45.4)	432 (44.6)	323 (37.8)	314 (43.3)	346 (46.8)

Abbreviation: SE, standard error.

^a^ Available for children aged 13–15 who answered “adequate” to the question that “Do you have adequate daily sleep time (adequate, slightly inadequate, and inadequate)?.”

Table 3 shows the cross‐sectional associations between dietary habit scores and sleep duration (2014–2019). The dietary habit scores were positively associated with sleep time for all groups of children, including the 4907 children who only attended one survey during 2014 and 2019. A one‐unit increase in dietary habit score was associated with a 0.018‐h increase in sleep duration. For children who attended two or more annual surveys, a significant longitudinal association was observed only in the age‐ and sex‐adjusted model, but not in the multivariable‐adjusted models (Table 4).

**TABLE 3:** Cross‐sectional associations of dietary habits with sleep time in Japanese children, the Fukushima Health Management Survey 2014–2019.

Dietary habit	Age group (year)	*n*	Dietary habit score, mean (SE)	Sleep duration (h), mean (SE)	Coefficient (95% CI)		
					Model 1	Model 2	Model 3
Score‐5	1–15	28,429	3.0 (0.01)	9.2 (0.01)	0.020 (0.013 to 0.027)^†^	0.018 (0.011 to 0.025)^†^	—
Score‐9	4–15	24,039	5.8 (0.01)	8.8 (0.01)	0.020 (0.013 to 0.026)^†^	0.018 (0.012 to 0.024)^†^	0.018 (0.012 to 0.025)^†^
Score‐10	7–15	18,639	6.7 (0.01)	8.4 (0.01)	0.019 (0.012 to 0.026)^†^	0.017 (0.010 to 0.024)^†^	0.018 (0.011 to 0.024)^†^
Score‐11	13–15	5885	6.5 (0.03)	7.3 (0.02)	0.029 (0.014 to 0.044)^†^	0.023 (0.009 to 0.038)^‡^	0.024 (0.010 to 0.038)^‡^

*Note:* Model 1 was adjusted for age (category) and sex (boy/girl); Model 2 was further adjusted for survey year (2014–2019), body mass index standard deviation score, current heath condition (very good, good, or normal/poor), physical activity (every day, two to four times a week, once a week, or none), and evacuation area (no/yes) for children aged ≥ 2 years; Model 3 was further adjusted for overall mental health problem (no/yes) and Strengths and Difficulties Questionnaire (continuous) for children aged ≥ 4 years.

Abbreviation: CI, confidence interval.

^†^ *p* < 0.001.

^‡^ *p* < 0.01.

**TABLE 4:** Longitudinal associations of changes in dietary habit scores with changes in sleep duration in Japanese children who attended two or more annual surveys, the Fukushima Health Management Survey 2014–2019.

Dietary habit	Age at baseline (years)	*n*	Change in dietary habit score, mean (SE)	Change in sleep duration (h), mean (SE)	Coefficient (95% CI)		
					Model 1	Model 2	Model 3
Score‐5	1–14	23,514	0.03 (0.01)	−0.6 (0.01)	0.013 (0.002 to 0.024)^‡^	0.005 (−0.004 to 0.015)	—
Score‐9	4–14	19,808	0.02 (0.01)	−0.5 (0.01)	0.018 (0.008 to 0.028)^†^	0.006 (−0.003 to 0.015)	0.007 (−0.002 to 0.015)
Score‐10	7–14	14,994	−0.03 (0.01)	−0.4 (0.01)	0.020 (0.009 to 0.031)^†^	0.005 (−0.005 to 0.015)	0.005 (−0.005 to 0.015)
Score‐11	13–14	3669	−0.02 (0.02)	−0.1 (0.01)	−0.010 (−0.037 to 0.017)	−0.009 (−0.036 to 0.018)	−0.009 (−0.036 to 0.018)

*Note:* Model 1 was adjusted for dietary habit score (continuous), age (category), and sleep duration at baseline and sex (boy/girl); Model 2 was further adjusted for survey years (2014–2019), body mass index standard deviation score (continuous), current health condition (very good, good, or normal/poor), physical activity (every day, two to four times a week, once a week, or none), and evacuation area (no/yes) for children aged ≥ 2 years; Model 3 was further adjusted for overall mental health problem (no/yes) and Strengths and Difficulties Questionnaire (continuous) for children aged ≥ 4 years.

Abbreviation: CI, confidence interval.

^†^ *p* < 0.001.

^‡^ *p* < 0.05.

For the question on self‐assessed adequate sleep time for junior high school students, the OR (95% CI) of score‐11 was 1.06 (1.02–1.09) (*p* < 0.001). This suggests that healthier dietary habits may lead to increased adequate sleep time in junior high school students. Meanwhile, children with inadequate sleep had a lower sleep duration (mean = 7.1 h, SE = 0.05) than those with adequate sleep (mean = 7.7 h, SE = 0.06) (*p* < 0.001).

The sensitivity analysis using the complete case analysis without imputations showed similar significant associations. Children with higher dietary habit scores had longer sleep durations than those with lower dietary habit scores (Table S4). Changes in dietary habit score‐5, score‐9, and score‐10 were significantly associated with changes in sleep duration in the age‐ and sex‐adjusted model, but not in the models further adjusted for additional variables (Table S5). Additionally, the OR (95% CI) for junior high school students on self‐assessed adequate sleep time associated with dietary score‐11 in the complete case analysis was 1.06 (1.03–1.10) (*p* < 0.001).

In the stratified analyses by evacuation status and overall mental health problems, the cross‐sectional associations between dietary habit scores and sleep duration were generally consistent with the main findings for each dietary habit score (Table 5). In contrast, the longitudinal associations between changes in dietary habit score and changes in sleep duration were weaker and less consistent across strata, particularly after adjustment for baseline sleep duration and follow‐up time. Only the interaction between evacuation status and dietary habit score‐5 was statistically significant.

**TABLE 5:** Associations of dietary habit scores with sleep duration in Japanese children by evacuation area and overall mental health problem, the Fukushima Health Management Survey 2014–2019.

Analysis	Stratification variable		Dietary habit score	Age group (years)	Coefficient (95% CI)	*p* for interaction
Cross‐sectional	Evacuation	No	Score‐5	1–15	0.007 (−0.002 to 0.017)	0.001
			Score‐9	4–15	0.013 (0.004–0.022)^‡^	0.170
			Score‐10	7–15	0.015 (0.006–0.025)^‡^	0.791
			Score‐11	13–15	0.020 (0.000–0.041)	0.283
		Yes	Score‐5	1–15	0.030 (0.019–0.040)^†^	
			Score‐9	4–15	0.023 (0.014–0.033)^†^	
			Score‐10	7–15	0.019 (0.009–0.029)^†^	
			Score‐11	13–15	0.028 (0.008–0.048)^‡^	
	Overall mental health problem	No	Score‐5	1–15	—	—
			Score‐9	4–15	0.014 (0.007–0.021)^†^	0.388
			Score‐10	7–15	0.015 (0.007–0.022)^†^	0.456
			Score‐11	13–15	0.020 (0.004–0.036)^§^	0.995
		Yes	Score‐5	1–15	—	
			Score‐9	4–15	0.024 (0.011–0.037)^†^	
			Score‐10	7–15	0.023 (0.009–0.037)^‡^	
			Score‐11	13‐15	0.027 (−0.001 to 0.056)	
Longitudinal	Evacuation	No	Score‐5	1–14	0.002 (−0.011 to 0.015)	0.147
			Score‐9	4–14	0.007 (−0.005 to 0.019)	0.644
			Score‐10	7–14	0.009 (−0.005 to 0.022)	0.921
			Score‐11	13–14	−0.009 (−0.047 to 0.030)	0.854
		Yes	Score‐5	1–14	0.010 (−0.005 to 0.026)	
			Score‐9	4–14	0.006 (−0.008 to 0.020)	
			Score‐10	7–14	0.000 (−0.014 to 0.015)	
			Score‐11	13–14	−0.010 (−0.048 to 0.027)	
	Overall mental health problem	No	Score‐5	1–14	—	—
			Score‐9	4–14	0.009 (−0.001 to 0.019)	0.322
			Score‐10	7–14	0.009 (−0.003 to 0.021)	0.265
			Score‐11	13–14	−0.008 (−0.041 to 0.024)	0.941
		Yes	Score‐5	1–14	—	
			Score‐9	4–14	0.000 (−0.018 to 0.018)	
			Score‐10	7–14	−0.004 (−0.023 to 0.015)	
			Score‐11	13–14	−0.011 (−0.060 to 0.039)	

*Note:* In the cross‐sectional analysis, models were adjusted for age (category), sex (boy/girl), survey years (2014–2019), body mass index standard deviation score (continuous), current health condition (very good, good, or normal/poor), physical activity (every day, two to four times a week, once a week, or none), and evacuation area (no/yes, omitted from the corresponding stratified model) for children aged ≥ 2 years; and overall mental health problem (no/yes, omitted from the corresponding stratified model) and Strengths and Difficulties Questionnaire (continuous) for children aged ≥ 4 years. In the longitudinal analysis, models were adjusted for age (category), sleep duration (continuous), dietary habit score (continuous), body mass index standard deviation score (continuous), current health condition (very good, good, or normal/poor), physical activity (every day, two to four times a week, once a week, or none), and evacuation area (no/yes, omitted from the corresponding stratified model) at baseline survey, sex (boy/girl), and follow‐up years (2014–2019) for children aged ≥ 2 years; and overall mental health problem (no/yes, omitted from the corresponding stratified model) and Strengths and Difficulties Questionnaire (continuous) for children aged ≥ 4 years.

Abbreviation: CI, confidence interval.

^†^ *p* < 0.001.

^‡^ *p* < 0.01.

^§^ *p* < 0.05.

## 4 Discussion

Significantly positive associations were observed between dietary habits and daily sleep duration in the cross‐sectional analysis, whereas the longitudinal analysis provided limited evidence of an association over time in Japanese children. Notably, the average effect of dietary habits on sleep time in this study population was small. It is relevant from a public health perspective, but the clinical significance may not be sufficient. Current publications have focused more on the association between insufficient sleep time and dietary habits [26]. The lack of evidence on the effect size of changes in sleep duration due to variant dietary habits in children makes it difficult to compare.

In our study, dietary habits and sleep duration were associated cross‐sectionally, whereas longitudinal analyses provided limited evidence that changes in dietary habits were associated with changes in sleep duration. The attenuation of the association after adjustment for follow‐up time suggests that the initial longitudinal findings may partly reflect age‐related declines in sleep duration. As children grew older, sleep duration generally decreased, with the magnitude of this reduction depending on the length of follow‐up. After accounting for this time‐related change, improvements in dietary habits were not clearly associated with longer sleep duration. The cross‐sectional association, consistent with most previous studies [7], may therefore reflect shared lifestyle patterns, family environment, regular daily routines, or overall health behaviors. Accordingly, the longitudinal findings should be interpreted cautiously, as they do not provide strong evidence of an independent effect of dietary improvement on sleep duration. Similar to findings in Danish children [27], the estimated effect was small and may be more relevant at the population level than at the individual level, with limited clinical significance.

Previous studies reported that children who consumed fruits and vegetables more frequently were more likely to achieve adequate overnight sleep, whereas soda and fast‐food consumption were associated with shorter overnight sleep [7, 14, 28]. A recent multipopulation study found that Mediterranean dietary patterns were associated with longer sleep duration and higher odds of sufficient sleep and reported potential causal associations between specific foods and sleep outcomes [29]. Studies highlighted that fruits and vegetables benefit sleep quality due to their anti‐inflammatory functions [30, 31]. Anti‐inflammatory diets may help improve sleep by promoting melatonin production, which is regulated by dark light and linked to the sleep rhythm [32]. In addition, higher fruit and vegetable consumption may improve dietary quality, reducing processed foods and snacking, which are related to lower sleep quality [30, 32]. Japanese diets are rich in fruits and vegetables, and our study results support this rationale on the association between healthy diets and sleep duration.

Extensive evidence suggests a bidirectional relationship between dietary intake and sleep patterns [7, 33]. An evening preference is associated with later mealtimes, breakfast skipping, and larger evening meals [34]. A Mendelian randomization study suggested that morning preference may increase the intake of healthy foods, particularly, fresh fruits and bran cereals [35]. Conversely, studies have reported that later bedtimes in children are associated with higher consumption of ultraprocessed and noncore foods [28], sugar and carbohydrates [36], and total energy intake [37, 38]. Nevertheless, when implementing research and interventions, the bi‐directional diet‐sleep relationships should be considered [39, 40].

The American Academy of Sleep Medicine and the Japanese Ministry of Health, Labour and Welfare (MHLW) recommend that a sleep duration of 11–14 h for toddlers (1–2 years), 10–13 h for preschool (3–5 years), 9–12 h for school‐age (6–12 years) (including naps), and 8–10 h for teenagers (13–18 years) regularly can promote optimal health [41, 42]. Similarly, in the Asia‐Pacific consensus statement, the recommended sleep durations are 11–14 h for toddlers (1–2 years), 10–13 h for preschoolers (3–4 years) [43], 9–11 h for 5–13 years, and 8–10 h for 14–18 years [44]. Japanese children do not have enough sleep time [16], and only 9% of 5‐year‐old Japanese children can achieve the recommended daily sleep [43]. In our study, Japanese school‐aged children had shorter daily sleep durations than recommended by the age‐specific MHLW and international guidelines.

A nationwide survey of 87,548 children reported that the total sleep times were 9.2, 9.1, 9.0, 8.7, 8.5, and 8.3 h/day in first‐, second‐, third‐, fourth‐, fifth‐, and sixth‐grade elementary children and 7.6, 7.4, 7.2 h/day in first‐, second, and third‐grade junior high school students [16]. In our study, the total sleep times were 9.2, 9.1, 9.0, 8.8, 8.7, and 8.4 h in elementary school children and 7.6, 7.3, and 7.1 h in junior high school children. The nationwide survey results had additional information on “time in bed,” approximately 0.3 h more than the sleep time. However, we did not have this information. Therefore, the total sleep time in our study might be lower than the national average. In addition, in the FHMS, the total daily sleep time included the nocturnal sleep duration and daytime nap for children under 6 years. However, significant associations were not observed between dietary habits and sleep duration for nocturnal and daytime naps, respectively (data not shown).

Inadequate sleep is related to immunity [45], childhood obesity [26, 46], and hypertension [47]. Shorter sleep duration and later meal timing are associated with adiposity gains in preschoolers [48]. The Japan National Health and Nutrition Survey reported that approximately 7.9% of children aged 6–14 had obesity in 2019 and 6.5% in 2018 [49]. Japanese breakfast (rice and miso soup) was associated with good eating and sleeping among pre‐ and elementary‐school children [50]. As a child grows, more chances of meals outside the home or family setting might lead to more irregular eating patterns, including breakfast skipping and late‐night eating [1, 51]. The positive association between health‐promoting behaviors and adequate sleep in adolescents observed in our study supports previous findings [52]. To control and prevent the non‐communicable disease burden, it is cost‐effective to promote health by cultivating healthy behaviors in children's early life [38, 43].

The 2011 FHMS showed that children with < 6 or > 12 h sleep duration had higher SDQ scores, and children aged 4–12 who moved to other prefectures had a longer sleep time [18]. The magnitude 3.11 earthquake and subsequent nuclear accidents could cause long‐term anxiety among the residents [18]. Sleep issues at an earlier age may predict behavioral issues during primary school [53, 54]. Nevertheless, continuous monitoring of children's sleep practices should keep pace with mental health issues.

The current study had several strengths. First, the large sampled data across comprehensive children's age groups and long follow‐up years clearly showed the sleeping status and examined its associations with dietary habits. Second, the longitudinal analysis enabled us to examine whether within‐child changes in dietary habits were associated with changes in sleep duration, an association that has been investigated in relatively few prospective studies. Third, the sensitivity analysis results using complete data were consistent with those obtained by multiple imputations. Fourth, numerous adjustments were made to the regression analysis, including mental health information from the measurement by SDQ and the parents' overall judgment of mental health issues in children.

The current study has some limitations. First, sleep duration was self‐ or proxy‐reported in the questionnaire, and the information on the discrepancy between time in bed (from bedtime to wake time) and actual sleep duration was not collected. Without objective measurements using methods such as actigraphy, reported sleep duration may be subject to recall error [25]. Second, the questions on dietary and behavioral habits had not been validated and were dichotomised into simple yes/no categories. They did not quantify food or nutrient intake using 24‐h dietary recalls or structured food‐frequency questionnaires [27]; thus, they might not capture the overall profile of children's dietary behavior. Such nondifferential misclassification may have biased the estimates toward the null and contributed to the small effect sizes observed in this study. Third, we did not collect data on screen time and the intensity of physical activity, which are important factors in evaluating sleep duration [55]. The behavioral pattern scores might be better than the simple combined categories [10, 56], but were not available for estimation due to the limited questions in our study. Further, we did not collect information on the perinatal and socioeconomic conditions of the family for adjustment in the analysis, which might have affected the significant associations [57, 58]. Therefore, residual confounding cannot be excluded, and unmeasured lifestyle and socioeconomic factors may have partly contributed to the observed associations.

## 5 Conclusions

In summary, healthy dietary habits were positively associated with sleep duration cross‐sectionally in Japanese children, particularly, schoolchildren, whereas longitudinal evidence was limited. The small effect size in this study may lack clinical significance, but from a public health perspective, it may be meaningful. In this regard, education on promoting healthy sleep through healthy dietary habits should be encouraged in schools, communities, and among family caregivers. Further prospective studies that include information on screen time and socioeconomic factors are needed to clarify the temporal relationships, underlying mechanisms, and magnitude of the associations between healthy lifestyle behaviors and sleep. Intervention studies may also help assess the replicability and practical relevance of these findings.

## Author Contributions

E.M., T.O., and M.H. designed the study. E.M. analyzed the data and drafted the manuscript. S.I., I.M., R.M., N.H., M.M., H.Y., H.T., M.H., and S.Y. collected, curated, and verified the data. T.O., M.M., H.Y., M.S., M.H., and S.Y. managed the surveys and supervised the research. T.O., M.M., S.I., I.M., H.T., R.M., N.H., M.S., M.H., and S.Y. interpreted data analysis and reviewed and edited the manuscript. All authors read and approved the final manuscript.

## Funding

This survey was conducted as part of the postdisaster recovery plans of Fukushima Prefecture. It was supported by the National Health Fund for Children and Adults Affected by the Nuclear Incident, Ministry of the Environment, Japan (MOEJ).

## Conflicts of Interest

The authors declare no conflicts of interest.

## Supporting information


**Table S1:** Dietary habit questions for Japanese children and adolescents, the Fukushima Health Management Survey 2014–2019.
**Table S2:** Missing numbers (%) of dietary habit data for imputations, the Fukushima Health Management Survey 2014–2019.
**Table S3:** Means (SDs) sleep duration by age compared with guideline recommendations, the Fukushima Health Management Survey 2014–2019.
**Table S4:** Cross‐sectional associations between dietary habit scores and daily sleep duration in Japanese children, the Fukushima Health Management Survey 2014–2019 (complete case analysis).
**Table S5:** Longitudinal associations between dietary habit scores and daily sleep duration in Japanese children who attended two or more annual surveys, the Fukushima Health Management Survey 2014–2019 (complete case analysis).

## Data Availability

The datasets generated and/or analysed during the current study are not publicly available because of confidentiality but are available from the corresponding author upon reasonable request and approval from the research committees.
