# Supplementary material for: Associations Between Dietary Habits and Sleep Duration in Japanese Children: The Fukushima Health Management Survey
Source: Pediatr Int. 2026 Oct 6;68(1):e70565. doi: 10.1111/ped.70565 (PMC13640044; doi:10.1111/ped.70565)
Supplement: Supplementary file 1 [file PED-68-e70565-s001.docx]

**Supplementary Table 1**. Dietary habit questions for Japanese children and adolescents, the Fukushima Health Management Survey 2014–2019.

| Questions: In the last month, _______________ | Code | 1–3 years | 4–6 years | 7–12 years | 13–15 years |
| --- | --- | --- | --- | --- | --- |
| 1. Did you/your child eat fish or fish foods ≥ 3 times/week? | No=0/Yes=1 | ✓ | ✓ | ✓ | ✓ |
| 2. Did you/your child eat unsalted vegetables, seaweed, or mushrooms daily? | No=0/Yes=1 | ✓ | ✓ | ✓ | ✓ |
| 3. Did you/your child eat fruits every day? | No=0/Yes=1 | ✓ | ✓ | ✓ | ✓ |
| 4. Did you/your child eat bean products (bean curd, deep-fried, fermented, boiled, etc.) daily? | No=0/Yes=1 | ✓ | ✓ | ✓ | ✓ |
| 5. Did you/your child eat dairy foods (milk, yogurt, etc.) daily? | No=0/Yes=1 | ✓ | ✓ | ✓ | ✓ |
| 6. Did you/your child drink sugared beverages (juice, sodas) daily? | Yes=0/No=1 | – | ✓ | ✓ | ✓ |
| 7. Did you/your child eat side/cooked foods (including instant foods) daily? | Yes=0/No=1 | – | ✓ | ✓ | ✓ |
| 8. Did you/your child eat meals faster than others? | Similar or Fast=0/Slow=1 | – | ✓ | ✓ | ✓ |
| 9. Did you/your child dine out every day? | Yes=0/No=1 | – | ✓ | ✓ | ✓ |
| 10. Did you/your child have breakfast every day? | No=0/Yes=1 | – | – | ✓ | ✓ |
| 11. Did you/your child go to bed within 1–2 hours after dinner? | No=0/Yes=1 | – | – | – | ✓ |

**Supplementary Table 2**. Missing number (%) of dietary habit data for imputations, the Fukushima Health Management Survey 2014–2019.

| Questions on dietary habits | 1–3 years  (n=4390) | 4–6 years  (n=5402) | 7–12 years  (n=12,752) | 13–15 years  (n=5885) | All  (n=28,429) |
| --- | --- | --- | --- | --- | --- |
| Eat fish or fish products more than three times/week | 21 (0.5) | 2 (0.04) | 12 (0.1) | 9 (0.2) | 44 (0.2) |
| Eat unsalted vegetables, seaweeds, or mushrooms every day | 17 (0.4) | 1 (0.02) | 10 (0.1) | 4 (0.1) | 32 (0.1) |
| Eat fruits every day | 20 (0.5) | 4 (0.1) | 12 (0.1) | 7 (0.1) | 43 (0.2) |
| Eat bean products (bean curd, deep fried, fermented, boiled, etc.) daily | 18 (0.4) | 4 (0.1) | 13 (0.1) | 10 (0.2) | 45 (0.2) |
| Eat dairy foods (milk, yogurt, etc.) every day | 17 (0.4) | 4 (0.1) | 11 (0.1) | 11 (0.2) | 43 (0.2) |
| Drink sugared beverages (juice, sodas) daily | – | 15 (0.3) | 11 (0.1) | 8 (0.1) | 34 (0.1) |
| Eat side/cooked foods (including instant foods) daily | – | 16 (0.3) | 10 (0.1) | 6 (0.1) | 32 (0.1) |
| Eat meals faster than others | – | 17 (0.3) | 29 (0.2) | 12 (0.2) | 58 (0.2) |
| Dine out every day | – | 14 (0.3) | 9 (0.1) | 7 (0.1) | 30 (0.1) |
| Have breakfast every day | – | – | 40 (0.3) | 2 (0.03) | 42 ( 0.2) |
| Go to bed within 1–2 hours after dinner | – | – | – | 39 (0.7) | 39 (0.7) |
| Current health condition | 61 (1.4) | 170 (3.2) | 530 (4.2) | 117 (2.0) | 878 (3.1) |
| Physical activity ^†^ | 31 (1.0) | 23 (0.4) | 41 (0.3) | 9 (0.2) | 104 (0.4) |
| SDQ ^‡^ | – | 15 (0.3) | 8 (0.1) | 47 (0.8) | 70 (0.3) |
| Overall mental health problem ^‡^ | – | 27 (0.5) | 55 (0.4) | 99 (1.7) | 181 (0.8) |

^†^ Available for children aged ≥2 years; ^‡^ Available for children aged ≥4 years. SDQ, Strengths and Difficulties Questionnaire.

**Supplementary Table 3**. Means (SDs) sleep duration by age compared with guideline recommendations, the Fukushima Health Management Survey 2014–2019.

| Age (year) | *n* | Fukushima  (hour) | Japanese recommendation^†^ (hour) | American consensus^‡^  (hour) | Asia-Pacific consensus^*^  (hour) |
| --- | --- | --- | --- | --- | --- |
| 1 | 1407 | 12.0 (0.9) |  |  |  |
| 2 | 1464 | 11.5 (0.8) |  |  |  |
| ***1-2*** | ***2871*** | ***11.8 (0.9)*** | ***11-14*** | ***11-14*** | ***11-14*** |
| 3 | 1519 | 11.1 (0.9) |  |  |  |
| 4 | 1649 | 10.6 (0.8) |  |  |  |
| ***3-4*** | ***3168*** | ***10.8 (0.9)*** |  |  | ***10-13*** |
| 5 | 1851 | 10.2 (0.8) |  |  |  |
| ***3-5*** | ***5019*** | ***10.6 (0.9)*** | ***10-13*** | ***10-13*** |  |
| 6 | 1902 | 9.8 (0.8) |  |  |  |
| 7 | 2075 | 9.2 (0.5) |  |  |  |
| 8 | 2145 | 9.1 (0.5) |  |  |  |
| 9 | 2146 | 9.0 (0.6) |  |  |  |
| 10 | 2176 | 8.8 (0.6) |  |  |  |
| 11 | 2095 | 8.7 (0.6) |  |  |  |
| 12 | 2115 | 8.4 (0.7) |  |  |  |
| ***6-12*** | ***14,654*** | ***9.0 (0.7)*** | ***9-12*** | ***9-12*** |  |
| 13 | 2077 | 7.5 (0.9) |  |  |  |
| ***5-13*** | ***18,582*** | ***8.9 (1.0)*** |  |  | ***9-11*** |
| 14 | 2024 | 7.3 (1.0) |  |  |  |
| 15 | 1784 | 7.1 (1.0) |  |  |  |
| ***13-15*** | ***5885*** | ***7.3 (1.0)*** | ***8-10*** | ***8-10*** |  |
| ***14-15*** | ***3808*** | ***7.2 (1.0)*** |  |  | ***8-10*** |

^†^Sleep Guide 2023. Ministory of Health, Labour and Welfare, Japan. <https://www.mhlw.go.jp/content/001305530.pdf> (accessed July 15, 2026). ^‡^Paruthi S et al. 2016. ^*^Loo BKG et al. 2023; Loo BKG et al. 2022;

**Supplementary Table 4**. Cross-sectional associations between dietary habit scores and daily sleep duration in Japanese children, the Fukushima Health Management Survey 2014–2019 (complete case analysis).

| Dietary habit | Age groups (year) | n | Dietary habit score,  Mean (SD) | Sleep duration (hour),  Mean (SD) | Coefficient (95% CI) | | |
| --- | --- | --- | --- | --- | --- | --- | --- |
|  |  |  |  |  | Model 1 | Model 2 | Model 3 |
| Score–five | 1–15 | 27,530 | 3.0 (1.38) | 9.2 (1.50) | 0.020 (0.013 to 0.027)^†^ | 0.018 (0.011 to 0.025)^†^ | - |
| Score–nine | 4–15 | 22,975 | 5.8 (1.61) | 8.8 (1.27) | 0.020 (0.013 to 0.026)^†^ | 0.018 (0.012 to 0.025)^†^ | 0.019 (0.013 to 0.026)^†^ |
| Score–ten | 7–15 | 17,794 | 6.6 (1.68) | 8.4 (1.06) | 0.019 (0.012 to 0.026)^†^ | 0.019 (0.012 to 0.026)^†^ | 0.019 (0.012 to 0.026)^†^ |
| Score–eleven | 13–15 | 5660 | 6.4 (1.78) | 7.3 (1.00) | 0.029 (0.014 to 0.043)^†^ | 0.026 (0.011 to 0.041)^†^ | 0.029 (0.014 to 0.044)^†^ |

Model 1 was adjusted for age (category) and sex (boy/girl); Model 2 was further adjusted for survey years (2014–2019), health condition (very good, good, or normal/poor), body mass index standard deviation score (continuous), physical activity (every day, 2–4 times a week, once a week, or none), and evacuation area (no/yes) for for children aged ≥2 years; and Model 3 was further adjusted for overall mental health problems (no/yes) and Strengths and Difficulties Questionnaire (continuous) for children aged ≥4 years. CI, confidential interval; ^†^*P* < 0.001.

**Supplementary Table 5**. Longitudinal associations between dietary habit scores and daily sleep duration in Japanese children who attended two or more surveys, the Fukushima Health Management Survey 2014–2019 (complete case analysis).

| Dietary habit | Age at baseline  (years) | n | Dietary habit score change  Mean (SD) | Sleep duration change (hour)  Mean (SD) | Coefficient (95% CI) | | |
| --- | --- | --- | --- | --- | --- | --- | --- |
|  |  |  |  |  | Model 1 | Model 2 | Model 3 |
| Score–five | 1–14 | 21,334 | 0.03 (1.12) | -0.6 (0.91) | 0.014 (0.003 to 0.025)^‡^ | 0.015 (-0.005 to 0.035) | - |
| Score–nine | 4–14 | 18,393 | 0.02 (1.26) | -0.5 (0.84) | 0.019 (0.009 to 0.029)^†^ | 0.011 (-0.004 to 0.027) | 0.012 (-0.003 to 0.027) |
| Score–ten | 7–14 | 13,878 | -0.03 (1.26) | -0.4 (0.75) | 0.020 (0.010 to 0.031)^†^ | 0.009 (-0.006 to 0.023) | 0.009 (-0.005 to 0.024) |
| Score-elevn | 13-14 | 3501 | -0.01 (1.18) | -0.1 (0.77) | -0.010 (-0.037 to 0.017) | -0.021 (-0.053 to 0.010) | -0.018 (-0.050 to 0.013) |

Model 1 was adjusted for age (category) and sex (boy/girl); Model 2 was further adjusted for survey years (2014–2019), health condition (very good, good, or normal/poor), body mass index standard deviation score (continuous), physical activity (every day, 2–4 times a week, once a week, or none), and evacuation area (no/yes) for for children aged ≥2 years; and Model 3 was further adjusted for overall mental health problems (no/yes) and Strengths and Difficulties Questionnaire (continuous) for children aged ≥4 years. ^†^*P* < 0.001; ^‡^*P* < 0.05.
